# *Postia alni* Niemelä & Vampola (Basidiomycota, Polyporales) – member of the problematic *Postia caesia* complex – has been found for the first time in Hungary

**DOI:** 10.3897/BDJ.2.e1034

**Published:** 2014-01-21

**Authors:** Viktor Papp

**Affiliations:** † Corvinus University of Budapest, Budapest, Hungary

**Keywords:** *Postia alni*, *Postia caesia* complex, *
Oligoporus
*, polypore, Hungary

## Abstract

Due to their bluish basidiocarps the *Postia caesia* (syn. *Oligoporus caesius*) complex forms a distinctive morphological group within the polypore genus *Postia* Fr., 1874. Five species of this group occur in Europe: *Postia alni* Niemelä & Vampola, *Postia caesia* (Schrad.) P. Karst., *Postia luteocaesia* (A. David) Jülich, *Postia mediterraneocaesia* M. Pierre & B. Rivoire and *Postia subcaesia* (A. David) Jülich. In this study *Postia alni* is reported for the first time from Hungary. The dichotomous key of the species of the European *Postia caesia* complex was prepared as well.

## Introduction

*Postia* Fr. is a brown rot polypore genus, which contains annual species with mainly soft, whitish basidiocarps, thin-walled, hyaline spores and monomitic hyphal system with clamped generative hyphae (Jülich 1982). Previously most of the taxa were considered to be members of the genus *Tyromyces* P. Karst. (e.g. Donk 1960, Jahn 1963, David 1974). However, Jülich (1982) proved that the type (*Tyromyces chioneus* (Fr.) P. Karst.) of *Tyromyces* P. Karst. is a white rot species. Hence he examined *Tyromyces* s. l. and found that *Postia* Fr. Fries, 1874 is the earlier legitimate name of the brown rot *Postia caesia* and *Postia subcaesia*. Ryvarden and Gilbertson combine for the same species into the genus *Oligoporus* Bref., 1888, based on the opinion, according to which *Postia* Fr. is a *nomen provisorium* or *nudum* of Fries (Gilbertson and Ryvarden 1985, Ryvarden and Gilbertson 1994). Nevertheless, the solution of Jülich that *Postia* was validly published by Fries (Fries 1874) was accepted by several mycologists (e.g. Larsen and Lombard 1986, Renvall 1992, Pegler and Saunders 1994). Previous phylogenetic study of *Tyromyces* s. l. shows that the distinction between *Postia* and *Oligoporus* is not significant (Yao et al. 1999). However a recent work shows that the two genera are different and the species of the *Postia caesia* complex belong to the *Postia* sensu stricto clade (Ortiz-Santana et al. 2013).

**Figure 2.**
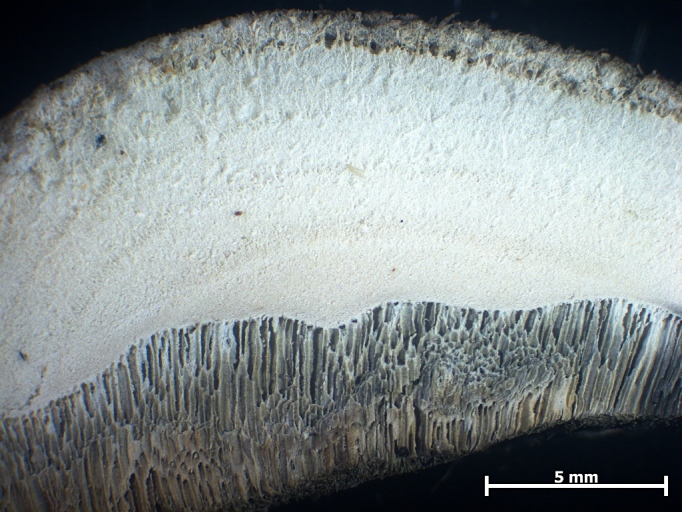
Cross-section of the basidiocarp of *Postia subcaesia*. Photo: V. Papp.

Based on the bluish tints of the basidiocarp and the lack of the chlamydospores in culture, *Postia caesia* complex forms a distinctive morphological group within the genus (Ţura et al. 2008). Five species of this group occur in Europe. *Postia caesia* (Schrad.) P. Karst. is a widespread species around the word, which in Europe grows preferably on gymnosperms (Bernicchia 2005, Ryvarden and Gilbertson 1994). *Postia subcaesia* (A. David) Jülich is macroscopically similar, but mainly grows on angiospermic trees and has narrower spores (Ryvarden and Gilbertson 1994). *Postia alni* Niemelä & Vampola also occurs on deciduous trees, but has smaller basidiocarp and the surface of the pileus is not as hairy as that of the *Postia subcaesia* has (Fig. 2) (Niemelä et al. 2001). *Postia luteocaesia* (A. David) Jülich is a rare Central-European species, which grows on *Pinus*. The main characteristic of this species is the bright yellow color of the basidiocarp besides the blue-grayish discoloration (Niemelä et al. 2004, Ryvarden and Gilbertson 1994). From the Mediterranean region *Postia mediterraneocaesia* M. Pierre & B. Rivoire has been described which has spores wider than 1.5 μm as *Postia caesia* and *Postia luteocaesia* (Pieri and Rivoire 2005).

Based on microscopic characters and host preference *Postia alni* shows a great similarity to *Postia subcaesia*. It differs by the matted pileus surface and the smaller size of the basidiocarp (Niemelä et al. 2001, Piątek 2003). Previously some mycologists (Enderle 1979, Jahn 1963, Vampola 1994) also observed the macroscopical variability of *Postia subcaesia* s. l., but there was not any valid new taxa name published. Velenovsky described a species (*Polyporus alni* Velen., 1922) which is identical with *Postia alni*, however it is illegitimate, because it is a later homonym of *Polyporus alni* Sorokin, 1892. Accordingly, Niemelä and Vampola described the new species under the name *Postia alni*, retaining the epithet, which was given by Velenovsky (Niemelä et al. 2001).

Previously in Hungary, only two species were known within the *Postia caesia* complex: *Postia caesia* and *Postia subcaesia* (e.g. Igmándy 1991, Szabó 2012). In this study *Postia alni* is recorded for the first time from Hungary from two locations.

## Materials and methods

**Figure 3.**
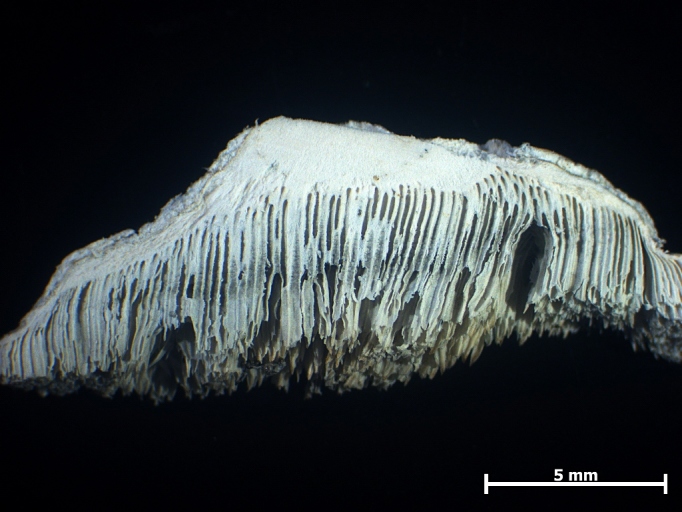
Cross-section of the basidiocarp of *Postia alni*. Photo: V. Papp.

The basidiocarps (Fig. 3) were collected in Juhdöglő-völgy Forest Reserve (Vértes Mts) and Dobogókő (Visegrádi Mts). Both of them were growing on dead *Fagussylvatica*. The specimens (PV188, PV977) were placed into the personal collection of the author (PV) and are available at the Botanical Department of Corvinus University of Budapest, Hungary. The identification of the specimens were based on the works of Niemelä et al. (2001) and Piątek (2003). For microscopical studies a Zeiss Axio Imager.A2 light microscope was used. For the measurements a × 1000 magnification objective, oil immersion and the program AxioVision Release 4.8.2 were used. The line drawings of the anatomical characteristics of the basidiocarp (Fig. 1) were made by the author with a drawing tube. The key of the European *Postiacaesia* complex was prepared after the following works: Bernicchia (2005), Niemelä et al. (2001), Niemelä et al. (2004), Pieri and Rivoire (2005) and Ryvarden and Gilbertson (1994).

## Taxon treatments

### Postia alni

Niemelä & Vampola, 2001

#### Materials

- **Type status:** Other material. **Occurrence:** recordNumber: PV 188; recordedBy: V. Papp; **Location:** continent: Europe; country: Hungary; county: Fejér; locality: Juhdöglő-völgy Forest Reserve; **Event:** year: 2010; month: 9; day: 14; habitat: on dead Fagus sylvatica; **Record Level:** institutionID: Corvinus University of Budapest
- **Type status:** Other material. **Occurrence:** recordNumber: PV 977; recordedBy: V. Papp; **Location:** continent: Europe; country: Hungary; county: Pest; locality: Dobogókő; **Event:** year: 2013; month: 11; day: 24; habitat: on dead Fagus sylvatica; **Record Level:** institutionID: Corvinus University of Budapest

#### Description

Basidiocarp annual, up to 3(–5) cm, white or cream color with bluish-grey tint. Pileus surface azonate, glabrous or slightly tomentose, but not fairy. Pores roundish, 4–5/mm. Context whitish, not zonate, soft when fresh, hard when dried. Hyphal system monomitic. Hyphae with clamp connections, thin- to thick-walled, 2.6–4.2 μm wide. Some contextual hyphae with finger-like branches. Cystidia absent and no cystidioles. Basidia clavate with 4 sterigmata and basal clamp, 10.2–15.6 μm. Basidiospores mostly allantoid, thin walled, 4.7–5.6 × 1.1–1.4 μm.

## Identification Keys

### Key to the European *Postia caesia* complex

**Table:** 

1	Basidiospores 1.5–2(2.2) μm wide, occurs on conifers or hardwoods	2
–	Basidiospores up to 1.5 μm wide, occurs mainly on hardwoods	4
2	Pore surface vivid yellow, basidiospores (4.5–)4.7–6.3(–6.5) × (1.5–)1.6–1.9(–2) μm (Q = 3.03–3.15), growing on *Pinus*, rare species	***Postia luteocaesia***
–	Bright yellow color not present	3
3	Mediterranean species, basidiocarps small size (up to 25 mm long), lightly greyish-blue when bruised, hyphae in pileipellis are encrusted, basidiospores (4,25–)4.7(–6.12) × (1.45–)1.5(–1.68) μm (Q = 3.2), occurs on conifers and hardwoods	***Postia mediterraneocaesia***
–	Wide spread species, basidiocarps larger (up to 6 cm long), upper surface tomentose to hairy, strongly bluish when bruised, basidiospores (4.4–)4.5–5.8(–6) × (1.3–)1.5–1.8(–2) μm, occurs mainly on conifers	***Postia caesia***
4	Basidiocarps orbicular, small, up to 4(5) cm, upper surface matted, or with very low tomentum, not hairy	***Postia alni***
–	Basidiocarps wide, larger, usually more than 5 cm, upper surface hairy	***Postia subcaesia***

## Discussion

There are many difficulties related to the identification of the species of the *Postia caesia* complex. There are some confusing East-Asian collection data that cannot be identified as either *Postia alni* or *Postia subcaesia* (Ţura et al. 2008). For instance, Wei and Dai (2006) mentioned *Postia alni* from China and gave spore size (less than 4 μm) that is different from the European taxa (4.4–6 μm in Niemelä et al. 2001). Molecular data (Yao et al. 2005) showed that the taxonomy of this group is more complicated and further studies are needed for the identification of the species of the complex.

## Supplementary Material

XML Treatment for Postia alni

## Figures and Tables

**Figure 1a.**
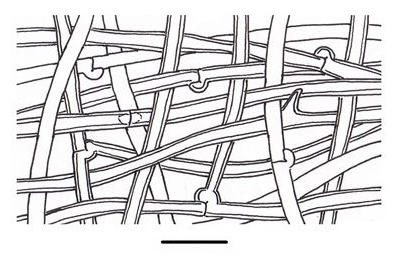
Contextual hyphal system;

**Figure 1b.**
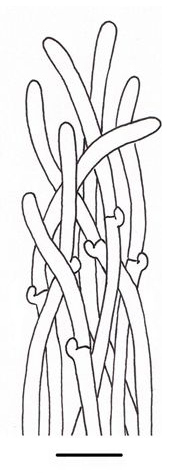
Hyphae from dissepiments edge;

**Figure 1c.**
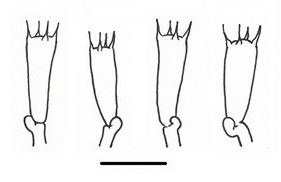
Basidia;

**Figure 1d.**
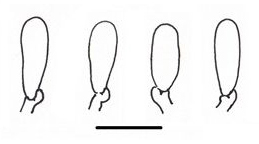
Basidioles;

**Figure 1e.**
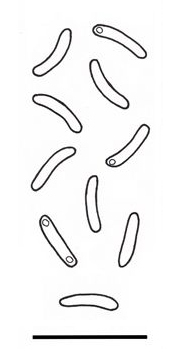
Basidiospores.
